# Clinical, Laboratory, and Histopathological Evaluation of 493 Patients Who Underwent Endoscopic Biopsy with a Presumptive Diagnosis of Celiac Disease: Association with Autoimmune Diseases

**DOI:** 10.5152/tjg.2023.22288

**Published:** 2023-07-01

**Authors:** Elife Kımıloğlu, Ahenk Karagülle, Meryem Keçeciler, Eylem Akbay Karatay, Deniz Koç

**Affiliations:** 1 Department of Pathology, Health Sciences University Faculty of Medicine, Gaziosmanpaşa Health Training and Research Center, İstanbul, Turkey; 2 Department of Pediatric Gastroenterology, Health Sciences University Faculty of Medicine, Gaziosmanpaşa Health Training and Research Center, İstanbul, Turkey; 3 Department of Gastroenterology, Health Sciences University Faculty of Medicine, Gaziosmanpaşa Health Training and Research Center, İstanbul, Turkey

**Keywords:** Autoimmune, biopsy, celiac disease, histopathology

## Abstract

**Background/Aims:**

Celiac disease is an immunological reaction provoked by gluten digestion in genetically vulnerable individuals in response to unknown environmental factors. It affects 0.7% of the world’s population and occurs at a rate of 1% in most nations. We aimed to assess the clinical, laboratory, and histopathological characteristics of patients with a presumable diagnosis of celiac disease and to investigate the coexistence of autoimmune disorders.

**Materials and Methods:**

In this retrospective study, data were gathered from the medical files of a total of 493 individuals with a preliminary diagnosis of celiac disease who underwent endoscopic biopsies. Analysis was carried out for clinical, biochemical, and histological results, as well as the presence of autoimmune disease.

**Results:**

Per the results of serological tests used in the diagnosis of celiac disease in this series, gliadin IgA and IgG positivities were found in 33.7% (n = 54/160) and 39.4% (n = 69/175) of patients; endomysium IgA and IgG positivities were detected in 37% (n = 88/238) and 18% (n = 30/167) of patients, while tissue transglutaminase IgA and IgG positivities were detected in 47.3% (n = 115/243) and 16.3% (n = 15/92) of patients, respectively. The incidence of patients with a CD3 level of ≥30% was 69.1% in 152 patients whose CD3 levels were tested.

**Conclusion:**

The general public and healthcare professionals need to be more aware of the prevalence and many signs of celiac disease. There is still a need to conduct the necessary research in this area. By boosting awareness, early diagnosis, and diet, it will be possible to prevent symptoms and negative consequences.

**Main Points**

- In our study, there is a statistically significant difference between Marsh classification groups as for positivities for endomysium IgA and IgG.
- Tissue transglutaminase IgA and IgG levels differ significantly between different groups of the Marsh classification system.
- The development of technologies for detecting and monitoring gluten in foods for end-stage gluten exposure may broaden future clinical studies.
- There is a requirement for greater awareness and implementation of further trials on this topic.
- By increasing awareness, early diagnosis, and diet, symptoms and harmful effects of celiac disease may be attenuated.

## INTRODUCTION

Celiac disease (CD), which affects about 1% of most populations, has recently been classified as a global disease, impacting 0.7% of the world’s population.^1^ The rate of diagnosis is rising, although this is attributable to an increase in incidence rather than increased awareness and detection. Gluten consumption triggers an immunological response in vulnerable individuals in reaction to unknown environmental factors. The sickness manifests itself in a wide range of ways. These signs and symptoms might range from severe malabsorption to nonsymptomatic or moderately symptomatic presentations.^2^

The appearance of duodenal villous atrophy is required for diagnosis, and most patients have circulating antibodies to tissue transglutaminase. In children, European guidelines enable diagnosis without duodenal biopsy if acute symptomatic and serological criteria are met. Despite excellent gluten-free diet (GFD) treatment, a small percentage of patients suffer persistent or recurrent symptoms. Adherence to a GFD is not always easy and such difficulties have prompted the employment of nondietary therapies, some of which are being tested on humans. Celiac disease is an autoimmune disease that arises in genetically susceptible individuals as a result of an immunological reaction to gluten.^2^

The seronegative type of CD exists in a small percentage of patients with CD villous atrophy but has negative specific serology. The histological reaction to GFD is used to diagnose this illness although other kinds of enteropathy not caused by gluten digestion must be ruled out.^3^ Patients with villous atrophy who are CD serology negative should not be referred to GFD unless alternative reasons have been extensively investigated. The seronegative CD is encountered in about 30% of cases with serology-negative villous atrophy.^3^

The diagnosis of traditional CD is supported by cases with complete immunoglobulin A (IgA) deficiency but have immunoglobulin G (IgG) endomysial/tissue transglutaminase antibody positivity.^3^

Celiac disease and type 1 diabetes mellitus are 2 autoimmune disorders that share many symptoms. Celiac disease is considered to have a constant prevalence of 1%-2%, primarily in Northern and Western European populations. It is also common in various parts of North Africa, the Middle East, and India. Although it is extremely rare in Japan, the data in other East Asian nations are lacking. Type 1 diabetes is frequent in Scandinavia, Sardinia, and the United Kingdom, as well as Canada and New Zealand, while it is uncommon in China and Venezuela. These 2 diseases are frequently found in the same geographic areas, although there is only a partial overlap; for example, CD is more common in Sweden than Finland, whereas type 1 diabetes mellitus is the opposite. In monozygotic twins, type 1 diabetes mellitus develops at a rate of approximately 42%. The most important indicator that genes are involved in the etiology of diabetes and CD (about 75%-90%) is the development of CD.^4^

In this study, we aimed to determine the clinical, biochemical, and histological features of patients with a preliminary diagnosis of CD and sought the coexistence of autoimmune diseases.

## MATERIALS AND METHODS

### Study Design

This retrospective study was performed in the pathology, gastroenterology, and pediatric gastroenterology departments of our tertiary care center. The approval of the University of Health Sciences Turkey Gaziosmanpaşa Training and Research Hospital Ethics Committee had been obtained before the study (21/10/2020/183). Each clinician obtained informed consent from the patients.

The endoscopic biopsy materials supplied to our laboratory in the medical pathology department of our institution between 2008 and 2020 were reevaluated. With the clinical presumptive diagnosis of CD, a total of 493 endoscopic biopsy materials underwent repeated histopathological evaluation. In this regard, those who had CD3 immunohistochemically were also reevaluated. The available serological results of the patients were assessed.

### Statistical Analysis

Statistical Package for the Social Sciences program version 25.0 (IBM Corp., Armonk, NY, USA) was used for statistical analysis. Descriptive data were expressed as mean, standard deviation, median, number, and percentage. Kruskal–Wallis *H*-test and Mann–Whitney *U*-test were performed for analysis of quantitative differences between groups. On the other hand, chi-square tests (Pearson chi-square test, continuity correction, and Fisher’s exact test) were performed for the comparison of categorical variables between groups. The compatibility of diagnostic tests was assessed with the kappa test. The results were evaluated at the 95% confidence interval, and the level of significance was set at a *P* < .05.

## Outcome Parameters

Age, positivities for gliadin IgA and IgG, endomysium IgA and IgG, tissue transglutaminase IgA and IgG as well as the intensity of CD3 positivity in lymphocytes were considered during the analysis of data in our series.

## RESULTS

### Age and Serological and Histopathological Features

The mean age in our series (n = 493) was 29.64 ± 17.69 years with a median age of 27 (range: 15-42). According to the age groups, 26% of cases were ≤15 years; 24.1% were in the age group of 16-27; 26% were aged between 28 and 42 years, and 23.9% were ≥43 years.

As for the serological test results used in the diagnosis of CD, gliadin IgA and IgG positivities were detected in 33.7% (n = 54/160) and 39.4% (n = 69/175). Endomysium IgA and IgG positivities were noted in 37% (n = 88/238) and 18% (n = 30/167) of our patients, respectively. Tissue transglutaminase IgA and IgG positivities were detected in 47.35 (n = 115/243) and 16.3% (n = 15/92) cases. The rate of patients with CD3 levels ≥30% was 69.1% out of a total of 152 cases. Per the Modified Marsh classification performed by histopathological examination, 68.4% (n = 337) of our series were at stage 0, 7.3% (n = 36) were at stage 1, 1.6% (n = 8) were at stage 2, and 22.7% (n = 112) were at stage 3 (a,b,c) (Table 1). When the age and serological test results are examined according to the Marsh-Oberhuber staging system, no statistically significant difference was found in the age distribution of patients per the Marsh classification (*P* > .05).

As for the Marsh classification, gliadin IgA positivity was 9% in grade 0, 33% in grades 1 and 2, 39%, 69%, and 78% in grades 3a, 3b, and 3c, respectively. Gliadin IgG positivity was 12% in stage 0, 35% in stages 1 and 2, 50%, 86%, and 89% in stages 3a, 3b, and 3c, respectively. There was a statistically significant difference in the presence of gliadin IgA and IgG as for the Marsh classification (IgA ⟶ χ^2^ = 50.54; *P* ≤ .001; IgG ⟶ χ^2^ = 72.08, *P* ≤ .001) (Table 2). According to Marsh classification, CD3 positivity was not detected in stage 0 but was 88% in stages 1 and 2, 96%, 93%, and 100% in stages 3a, 3b, and 3c, respectively (χ^2^ = 122.27, *P* ≤ .001) (Table 2).

The sensitivity of the gliadin IgA serological test was 55.3%, the specificity was 90.7%, the positive predictive value (PPV) was 87%, the negative predictive value (NPV) was 64.2%, and the accuracy rate was 71.9% in the determination of the histological amplification (Marsh class 0 vs. Marsh class 1 and 3) when the level of compliance and reliability in determining the Modified Marsh classification of serological tests was examined. The gliadin IgA serological test and the Modified Marsh classification had a statistically significant agreement (*κ* = 0.467; *P* ≤ .001) (Table 3). The gliadin IgG serological test has a sensitivity of 65.6%, specificity of 88.2%, PPV of 85.5%, NPV of 70.8%, and accuracy of 76.6% in predicting histological staging (Marsh class 0 vs. Marsh classes 1 and 3). The gliadin IgG serological test and the Modified Marsh classification had a statistically significant agreement (*κ* = 0.541; *P* < .001) (Table 3).

The endomysium IgA serological test has a sensitivity of 67.8%, specificity of 91.9%, PPV of 88.6%, NPV of 75.3%, and an accuracy of 80.3% in detecting the histological staging (Marsh class 0 vs. Marsh classes 1 and 3). The endomysium IgA serological test and the Modified Marsh classification had a statistically significant agreement (*κ* = 0.591; *P* < .001) (Table 3). In detecting histological amplification (Marsh class 0 vs. Marsh classes 1 and 3), the endomysium IgG serological test had a sensitivity of 35.1%, a specificity of 96.7%, a PPV of 90%, an NPV of 63.5%, and an accuracy of 68.3%. The endomysium IgG serological test and the Modified Marsh classification had a statistically significant low degree of agreement (*κ* = 0.300; *P* < .001) (Table 3).

Tissue transglutaminase IgA serological test has a sensitivity of 75.7%, specificity of 78.1%, PPV of 75.7%, NPV of 78.1%, and accuracy of 77% in identifying histological amplification (Marsh class 0 vs. Marsh classes 1 and 3). The tissue transglutaminase IgA serological test and the Modified Marsh classification displayed a statistically significant agreement (*κ* = 0.535; *P* < .001) (Table 3). In detecting histological amplification (Marsh class 0 vs. Marsh classes 1 and 3), the sensitivity of the tissue transglutaminase IgG serological test was 28.9%, specificity 95.7%, PPV was 86.7%, NPV was 58.4%, and accuracy was 63%. The tissue transglutaminase IgG serological test and the Modified Marsh classification have a statistically significant low degree of agreement (*κ* = 0.243; *P* = .001) (Table 3). The sensitivity of lymphocyte CD3 high positivity (30%) in determining the histological staging (Marsh class 0 vs. Marsh classes 1 and 3) was 93.8%, the specificity was 100%, the PPV was 100%, the NPV was 85.1%, and the accuracy rate was 95.4%. There was a statistically significant agreement between CD3 high positivity and the Modified Marsh categorization (*κ* = 0.616; *P* < .001) (Table 3).

There was no statistically significant difference between the ages of the patients and their CD3 levels (Table 4). In serologically gliadin IgA- and IgG-positive patients, the rate of CD3 ≥30% positivity was statistically substantially greater than that in negative patients (*P* < .001) (Table 4). In serological endomysium, IgA- and IgG-positive individuals, the rate of CD3 ≥30% positivity was statistically substantially greater than in negative patients (for IgA, *P* = .008; for IgG, *P* = .001) (Table 4). The rate of CD3 ≥30% positivity was statistically substantially greater in tissue transglutaminase IgA- and IgG-positive patients compared to negative patients (*P* = .001 for IgA; *P* = .018 for IgG) (Table 4). CD3 ≥30% positivity rate was 0% in stage 0, 88.2% in stages 1 and 2, and 96.2% in stage 3 patients. As the stage increased in patients, CD3 ≥30% positivity rate was found to increase statistically significantly (χ^2^ = 121.97, *P* < .001) (Table 4).

**Figure 1.**
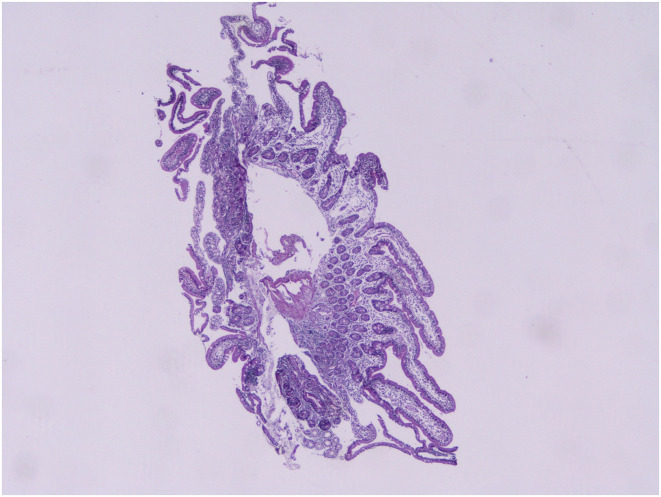
Villus and crypt structures are preserved, intraepithelial lymphocytosis >30/100 enterocytes, Marsh class 1, hematoxylin and eosin, 40×.

**Figure 2.**
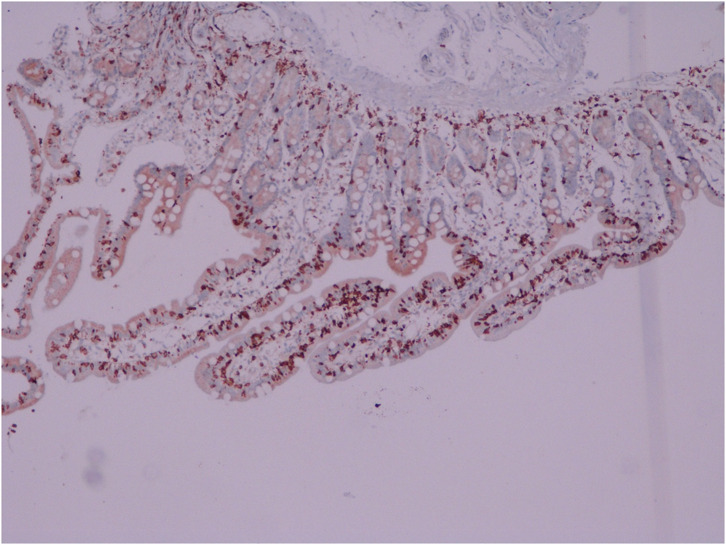
Villus and crypt structures are preserved, intraepithelial lymphocytosis >30/100 enterocytes, Marsh class 1, CD3 immunohistochemistry, 100×.

**Figure 3.**
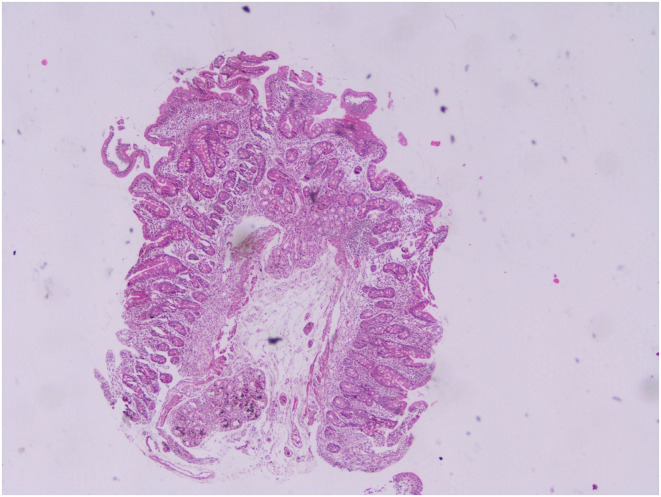
Mild degree atrophy of villi, intraepithelial lymphocytosis >30/100 enterocytes, Marsh class 3A, hematoxylin and eosin, 40×.

**Figure 4.**
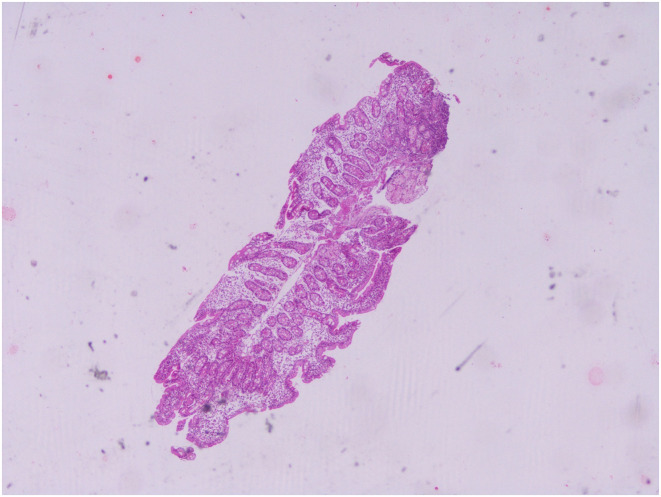
Moderate degree atrophy of villi, crypt hyperplasia, intraepithelial lymphocytosis >30/100 enterocytes, Marsh class 3B, hematoxylin and eosin 40×.

**Figure 5.**
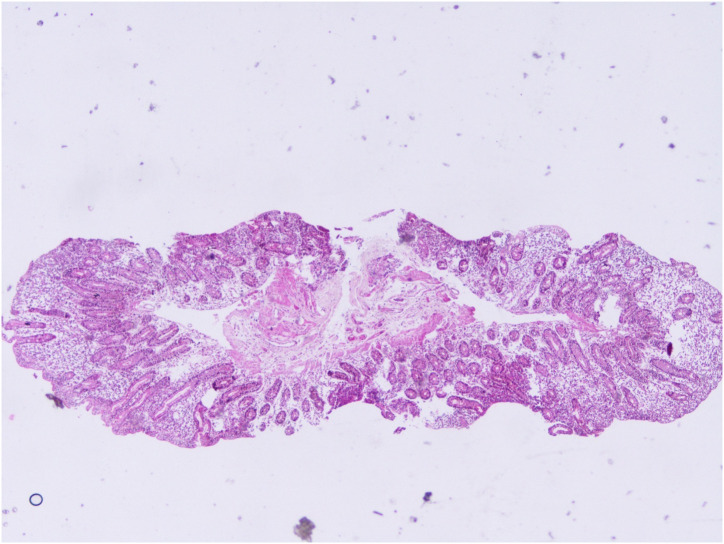
Severe degree atrophy of villi, crypt hyperplasia, intraepithelial lymphocytosis >30/100 enterocytes, Marsh class 3B, hematoxylin and eosin, 40×.

**Figure 6.**
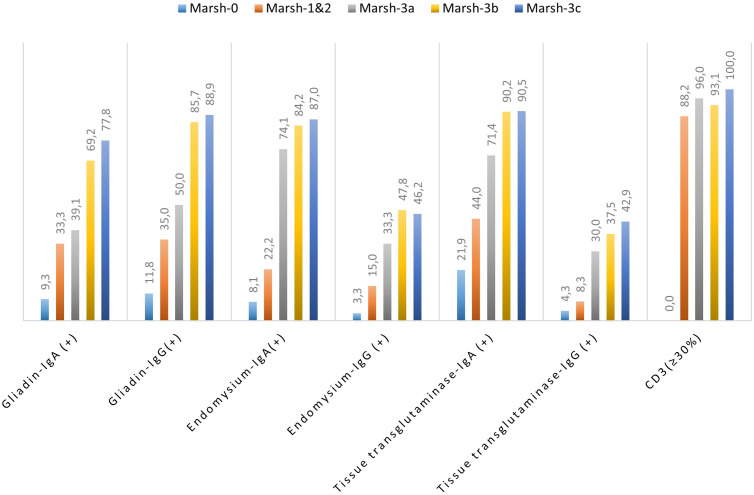
Graphic of serological test results per the Marsh–Oberhuber Staging System.

An overview of serological test results per the Marsh–Oberhuber Staging System has been shown in Figures 1-6 demonstrating the structural alterations in villi, crypts, and infiltration of lymphocytes at different stages of Marsh classification.

## DISCUSSION

Intraepithelial lymphocytes express the natural killer Tlymphocyte receptors NKG2D and CD9/NKG2A during the pathogenesis of CD, which detects products of the stress-induced genes and Human Leukocyte Antigen E (HLA-E) protein released from the epithelial cell surface.^2^ The upregulation of natural killer (NK) receptors on epithelial cells is aided by cytotoxicity.^2^ The creation of a complete pathological CD lesion appears to need both lamina propria (adaptive) and intraepithelial (innate) immune responses, although it is unclear how these 2 processes interact.^2^

Gluten should be introduced to an infant between the ages of 4 and 10 months, according to the European Society of Pediatric Gastroenterology, Hepatology, and Nutrition guidelines, and excessive doses of gluten should be avoided in the first weeks after gluten introduction.^2^ Gluten exposure after 6 months of CD increases the risk, according to a meta-analysis.^2^ Celiac disease is becoming more common around the world.^5^

Rare symptoms such as prolonged diarrhea, weight loss, and growth retardation are typical with CD. Iron deficiency, bloating, constipation, persistent fatigue, headache, stomach discomfort, and osteoporosis are the most prevalent nonclassical symptoms.^6-8^

Serological tests to show the existence of autoantibodies when the patient is on a regular gluten-containing diet, followed by gastroduodenoscopy and duodenal biopsies, are the last methods for diagnosing CD.^9,10^ Because of its high sensitivity and NPV, IgA-tTG (Tissue Transglutaminase) antibody concentration should be determined as the initial screening test. This antibody is less expensive than endomysial antibodies (EMA). The IgAtTG test is more sensitive for CD than the IgA-tTG and EMA tests taken separately. If IgA-tTG is weakly positive, EMA levels should be tested as well. Because false-negative results in patients with IgA deficiency are possible, total IgA concentration should be evaluated in conjunction with serology. IgG-tTG, IgG-EMA, and IgG-deamidegliadinpeptide can be measured in patients with IgA deficiency.^2^

Increased intraepithelial lymphocytes, crypt hyperplasia, and villous atrophy (Marsh type 3) in duodenal biopsy specimens with positive celiac serology validate the diagnosis of CD in adults.^2^ Despite following a GFD, over 20% of celiac patients may experience persistent or recurrent symptoms. Celiac disease with refractory symptoms despite the presence of persistent or recurring malabsorption symptoms and villous atrophy may be linked to an elevated risk of death and can lead to enteropathy-associated T-cell lymphoma.^11^ T cells produce tissue damage using interferon (IFN)-alpha, IFN-gamma, tumor necrosis factor (TNF), interleukin (IL)-4, IL-5, and IL-21, some of which have the ability to induce tissue damage.^9^ Celiac disease is linked to a variety of non-Hodgkin lymphomas, including enteropathy-associated T-cell lymphoma. Adenocarcinoma of the esophagus, small intestine, colon, liver, and pancreas was also found to be more common in a population-based investigation.^2^ Pathologies associated with CD include type 1 diabetes mellitus, selective IgA deficiency, Down syndrome, Turner syndrome, autoimmune thyroid and hepatic diseases, and dermatitis herpetiformis.^12^ The extra gastrointestinal manifestations of CD consist of irritability, fatigue, growth retardation, persistent iron deficiency anemia, vitamin D deficiency, aphthous stomatitis, delayed puberty, and osteoporosis.^1,4,13^

Although villous atrophy affects a small fraction of CD patients, particular serology is negative at the time of diagnosis, and they are classified as seronegative CD patients.^1,3^ These individuals should not be referred to GFD unless they have been thoroughly evaluated for other possible reasons. On the other hand, about 30% of patients with villous atrophy who do not have a positive serology have seronegative CD.^3^

When the serological test results used in the diagnosis of CD were examined in our study, gliadin IgA and IgG positivities were found in 33.7% (n = 54/160) and 39.4% (n = 69/175); endomysium IgA and IgG positivities were found in 37% (n = 88/238) and 18% (n = 30/167); tissue transglutaminase IgA and IgG positivities were found in 47.3% (n = 115/243) and 16.3% (n = 15/92). The rate of patients with a CD3 level of 30% and above in 152 patients who were evaluated as CD3 level was 69.1%. A statistically significant difference was noted between Marsh classification groups as for the positivities for endomysium IgA and IgG. Similarly, tissue transglutaminase IgA and IgG levels differed significantly between different groups of the Marsh classification system.

Celiac disease in adults is confirmed by duodenal biopsy specimens with increased intraepithelial lymphocytes, crypt hyperplasia, and villous atrophy (Marsh type 3), as well as positive serology for CD.^2^ Despite following a GFD, over 20% of celiac patients may experience persistent or recurrent symptoms. Malabsorption and villous atrophy are persistent or recurrent symptoms of refractory CD. This disorder is linked to a higher risk of death and can lead to enteropathy-associated T-cell lymphoma.^1,9^ T cells produce IFN-alpha, IFN-gamma, TNF, IL-4, IL-5, and IL-21, which can induce tissue damage in some cases.^9^ Coeliac disease is linked to a variety of non-Hodgkin lymphomas, including enteropathy-associated T-cell lymphoma. Adenocarcinoma of the esophagus, small intestine, colon, liver, and pancreas was also found to be more common in a population-based investigation.^2^

Coeliac disease and type 1 diabetes mellitus are 2 autoimmune diseases that share common genetic and immunological findings.^9,10,14^ Mild and asymptomatic forms of CD are often observed in children with type 1 diabetes mellitus. Systematic screening of children with type 1 diabetes mellitus for CD may increase the diagnostic yield, but this approach must be performed in a controlled manner. Further prospective, multicentric trials are warranted to screen children with type 1 diabetes mellitus who are asymptomatic for CD. The role of serology in CD is increasing rapidly and a number of novel pharmacological treatments are under development.^4^

Type 1 diabetes is most common in early adolescence, although CD can occur at any age. Furthermore, females are twice more likely to be affected by CD compared to males. Males are more likely than girls to develop type 1 diabetes, especially in high-risk countries.^6^ High concordance in the development of type 1 diabetes (about 42%) and CD (approximately 75%-90%) in monozygotic twins is the most important evidence demonstrating the role of genes in the etiology of diabetes mellitus and CD.^4^ The diagnosis of CD in children can be established without biopsy using high levels of tissue transglutaminase autoantibodies, endomysial autoantibodies, and CD-type HLA genotypes.^4,15^

As a consequence of the diagnostic efficacy of the antibodies and their compatibility with the biopsy results, The European Society for Paediatric Gastroenterology Hepatology and Nutrition (ESPGHAN) does not recommend the routine use of biopsy for CD in children in 2022.^16,17^ ESPGHAN guidelines in 2012 recommend TG-IgA testing, which is highly sensitive and specific, and less costly than EMA IgA antibody test as an initial screening test for suspected CD, and total IgA to rule out selective IgA deficiency. For children under 2 years of age, analysis of the deamidated gliadin peptide (DGP) IgA test is recommended. If IgA deficiency is present, tTG-IgG test or EMA-IgG test or DGP-IgG test should also be performed.^18^

If serological tests are negative for tTG-IgA and the total IgA level is normal, CD is unlikely. For individuals with low serum IgA levels (total serum IgA <0.2 g/L), the results of IgG class CD-specific antibody tests are evaluated. The reasons leading to a false-negative tTG result should also be considered. These factors involve low gluten intake, protein-losing enteropathy, use of immunosuppressive drugs, and patients under 2 years of age. If tTG is found positive (less than 10 times the upper limit of normal level), gastroduodenoscopy and multiple biopsies of the small intestine should be performed to confirm the diagnosis.^18^

If the tTG is greater than 10 times the upper limit of the normal level in a symptomatic patient, it should be discussed with parents to make a diagnosis of CD without biopsy. If parents agree, EMA test and HLA-DQ2/DQ8 analysis are performed. An EMA test is done from a second blood sample to rule out a false positive tTG test. If EMA and HLA-DQ2 or HLA-DQ8 are positive, the diagnosis of CD is made without biopsy.^18^

The 2020 ESPGHAN guidelines report that the combination of tTG-IgA testing and total IgA testing is more accurate than any other test combination as the initial test for suspected CD, regardless of age. If the total IgA level is found to be low, tTG-IgG test or EMA-IgG test or DGP-IgG test should be performed.^19^

Approximately 2%-3% of CD patients have negative IgA-based serological tests due to IgA deficiency.^20^ In our study, selective IgA deficiency was found in 5 patients under the age of 18 and in 10 patients aged >18 years. This data is also compatible with relevant literature.^20^

Anti-gliadin antibodies are not recommended for diagnosis due to their low sensitivity and specificity. However, the diagnostic value of second-generation antigliadin antibodies is higher than first-generation tests with 94% sensitivity and 97% specificity.^21,22^ Anti-gliadin antibodies may also be elevated in nonceliac gluten sensitivity. It has also been observed to be high in nonceliac gluten sensitivity and autoimmune diseases. In our study, it was requested because the gastrointestinal complaints of the patients were at the forefront.^23^

The main limitations of the present study include retrospective design, data restricted to the experience of a single-center, and possible confounding factors such as ethnicity and socioenvironmental factors. Thus, extrapolation of our data to larger populations must be made cautiously.

## CONCLUSION

The development of technologies for detecting and monitoring gluten in foods for end-stage gluten exposure may broaden future clinical studies and may have a significant impact on patient’s everyday activities. Other environmental factors, such as cesarean section ratios, iron supplementation during pregnancy, newborn feeding practice, infant and early childhood nutritional practice, and microbial diseases in patients with CD and autoimmune diseases should be investigated in future studies. There is a requirement for greater awareness and implementation of further trials on this topic. By increasing awareness, early diagnosis, and diet, symptoms and harmful effects of CD may be attenuated.

## Figures and Tables

**Table 1.** An Overview of Age, Serological, and Histopathological Variables in Our Series (n = 493).

Variable	Category	n	%
Age group	≤15	128	26.0
	16-27	119	24.1
	28-42	128	26.0
	≥43	118	23.9
Age group	<18	169	34,3
	≥18	324	65,7
Antigen–antibody type			
Gliadin-IgA (n = 160)	Negative	106	66.3
	Positive	54	33.7
Gliadin-IgG (n = 175)	Negative	106	60.6
	Positive	69	39.4
Endomysium-IgA (n = 238)	Negative	150	63.0
	Positive	88	37.0
Endomysium-IgG (n = 167)	Negative	137	82.0
	Positive	30	18.0
Tissue transglutaminase-IgA (n = 243)	Negative	128	52.7
	Positive	115	47.3
Tissue transglutaminase-IgG (n = 92)	Negative	77	83.7
	Positive	15	16.3
Lymphocyte-CD3 (%) (n = 152)	<30	47	30.9
	≥30	105	69.1
Marsh–Oberhuber Modified Marsh Classification	0	337	68.4
	1	36	7.3
	2	8	1.6
	3a	32	6.5
	3b	49	9.9
	3c	31	6.3

IQR, interquartile range; SD, standard deviation.

**Table 2.** Age and Serological Test Results Per Marsh–Oberhuber Staging System

Variables		**Modified Marsh Classification**					
	**0**	**1 and 2**	**3a**	**3b**	**3c**	**Significance**	
	n (%)	n (%)	n (%)	n (%)	n (%)	***K** **–** **W/χ** **^2^***	***P***
Age, mean (SD)	30.14 (17.78)	30.59 (15.58)	29.72 (17.48)	27.06 (19.95)	26.87 (16.38)	3.033^a^	.552
Age group						15.576^b^	.211
≤15	83 (24.6)	10 (22.7)	8 (25.0)	17 (34.7)	10 (32.3)		
16-27	91 (27.0)	8 (18.2)	5 (15.6)	10 (20.4)	5 (16.1)		
28-42	75 (22.3)	16 (36.4)	11 (34.4)	15 (30.6)	11 (35.5)		
≥43	88 (26.1)	10 (22.7)	8 (25.0)	7 (14.3)	5 (16.1)		
Age group						1.464	.833
<18	117 (34.7)	13 (29,5)	9 (28,1)	19 (38,8)	11 (35,5)		
≥18	220 (65,3)	31 (70,5)	23 (71,9)	30 (61,2)	20 (64,5)		
Gliadin-IgA (*n* = 160)						**50.540** **^b^**	<.001*
Negative	68 (90.7)	12 (66.7)	14 (60.9)	8 (30.8)	4 (22.2)		
Positive	7 (9.3)	6 (33.3)	9 (39.1)	18 (69.2)	14 (77.8)		
Gliadin-IgG (*n* = 175)						**72.080** **^b^**	<.001*
Negative	75 (88.2)	13 (65.0)	12 (50.0)	4 (14.3)	2 (11.1)		
Positive	10 (11.8)	7 (35.0)	12 (50.0)	24 (85.7)	16 (88.9)		
Endomysium-IgA (*n* = 238)						**123.424** **^b^**	<.001*
Negative	113 (91.9)	21 (77.8)	7 (25.9)	6 ((15.8)	3 (13.0)		
Positive	10 (8.1)	6 (22.2)	20 (74.1)	32 (84.2)	20 (87.0)		
Endomysium-IgG (*n* = 167)						**37.485** **^b^**	<.001*
Negative	87 (96.7)	17 (85.0)	14 (66.7)	12 (52.2)	7 (53.8)		
Positive	3 (3.3)	3 (15.0)	7 (33.3)	11 (47.8)	6 (46.2)		
Tissue transglutaminase-IgA (n = 243)						**85.876** **^b^**	<.001*
Negative	100 (78.1)	14 (56.0)	8 (28.6)	4 (9.8)	2 (9.5)		
Positive	28 (21.9)	11 (44.0)	20 (71.4)	37 (90.2)	19 (90.5)		
Tissue transglutaminase-IgG (*n* = 92)						**15.818** **^b^**	.003*
Negative	45 (95.7)	11 (91.7)	7 (70.0)	10 (62.5)	4 (57.1)		
Positive	2 (4.3)	1 (8.3)	3 (30.0)	6 (37.5)	3 (42.9)		
Lymphocyte-CD3 (%) (n = 152)						**122.265** **^b^**	<.001*
<30	40 (100.0)	4 (11.8)	1 (4.0)	2 (6.9)	0 (0.0)		
≥30	0 (0.0)	30 (88.2)	24 (96.0)	27 (93.1)	24 (100.0)		

*P < .05. Those with value of P ≤ .001 are made in bold and significant.

^a^K–W, Kruskal–Wallis *H* test; ^b^ *χ^2^*, chi-square test.

**Table 3.** Conformity and Reliability of Serological Tests to the Modified Marsh Classification

Antigen–Antibody Type				Sensitivity	Specificity	PPV	NPV	Accuracy
Gliadin-IgA	**1-3**	**0**	**Total**	55.3%	90.7%	87%	64.2%	71.9%
Negative	38	68	106					
Positive	47	7	54					
Total	85	75	160					
	***κ*** = **0.467; ** ***P*** ≤ .001*							
Gliadin-IgG	**1-3**	**0**	**Total**	65.6%	88.2%	85.5%	70.8%	76.6%
Negative	31	75	106					
Positive	59	10	69					
Total	90	85	175					
	***κ*** = **0.541;** *** P*** ≤ .001*							
Endomysium-IgA	**1-3**	**0**	**Total**	67.8%	91.9%	88.6%	75.3%	80.3%
Negative	37	113	150					
Positive	78	10	88					
Total	115	123	238					
	***κ*** = **0.591; ** ***P*** ≤ .001*							
Endomysium-IgG	**1-3**	**0**	**Total**	35.1%	96.7%	90%	63.5%	68.3%
Negative	50	87	137					
Positive	27	3	30					
Total	77	90	167					
	***κ*** = **0.300; ** ***P*** ≤ .001*							
Tissue transglutaminase-IgA	**Marsh-1-3**	**Marsh-0**	**Total**	75.7%	78.1%	75.7%	78.1%	77%
Negative	28	100	128					
Positive	87	28	115					
Total	115	128	243					
	***κ*** = **0.535; ** ***P*** ≤ .001*							
Tissue transglutaminase-IgG	**1-3**	**0**	**Total**	28.9%	95.7%	86.7%	58.4%	63%
Negative	32	45	77					
Positive	13	2	15					
Total	45	47	92					
	***κ*** = **0.243; ** ***P*** = .001*							
Lymphocyte-CD3 (%)	**1-3**	**0**	**Total**	93.8%	100%	100%	85.1%	95.4%
<30	7	40	47					
≥30	105	0	105					
Total	112	40	152					
	***κ*** = **0.616; ** ***P*** ≤ .001*							

*P < .05.

*κ*, Kappa test; NPV, negative predictive value; PPV, positive predictive value.

**Table 4.** CD3 Levels Per Serological Test Results and Marsh–Oberhuber Staging System

Variables	Lymphocyte-CD3			
	<30%	≥30%	Significance	
	*n* (%)	*n* (%)	*Z/χ^2^*	*P*
Age, mean (SD) (*n* = 152)	27.38 (15.97)	28.06 (17.37)	–0.136^a^	.892
Age group (*n* = 152)				
≤15	11 (27.5)	29 (72.5)	1.431^b^	.698
16-27	14 (38.9)	22 (61.1)		
28-42	14 (29.2)	34 (70.8)		
≥43	8 (28.6)	20 (71.4)		
Age group				
<18	15 (30.0)	35 (70.0)	0.000^c^	1.000
≥18	32 (31.4)	70 (68.6)		
Gliadin-IgA (n = 84)				
Negative	21 (43.8)	27 (56.3)	**-** **^d^**	<.001*
Positive	2 (5.6)	34 (94.4)		
Gliadin-IgG (*n* = 91)				
Negative	25 (55.6)	20 (44.4)	**-** **^d^**	<.001*
Positive	2 (4.3)	44 (95.7)		
Endomysium-IgA (*n* = 107)				
Negative	30 (55.6)	24 (44.4)	**-** **^d^**	<.001*
Positive	4 (7.5)	49 (92.5)		
Endomysium-IgG (*n* = 80)				
Negative	23 (37.7)	38 (62.3)	**-** **^d^**	.008*
Positive	1 (5.3)	18 (94.7)		
Tissue transglutaminase-IgA (*n* = 108)				
Negative	21 (50.0)	21 (50.0)	**10.792** **^c^**	.001*
Positive	12 (18.2)	54 (81.8)		
Tissue transglutaminase-IgG (*n* = 50)				
Negative	19 (45.2)	23 (54.8)	**-** **^d^**	.018*
Positive	0 (0.0)	8 (100.0)		
Modified Marsh classification (*n* = 152)				
0	40 (100.0)	0 (0.0)	**121.972** **^b^**	<.001*
1 & 2	4 (11.8)	30 (88.2)		
3a, 3b, 3c	3 (3.8)	75 (96.2)		

*P < .05; ^a^(Z), Mann–Whitney *U* Test; χ^2^, chi-square tests; ^b^Pearson chi-square test; ^c^Continuity correction, ^d^Fisher’s exact test.
